# The MAP Kinase SsKpp2 Is Required for Mating/Filamentation in *Sporisorium scitamineum*

**DOI:** 10.3389/fmicb.2018.02555

**Published:** 2018-10-26

**Authors:** Yi Zhen Deng, Bin Zhang, Changqing Chang, Yixu Wang, Shan Lu, Shuquan Sun, Xiaomeng Zhang, Baoshan Chen, Zide Jiang

**Affiliations:** ^1^State Key Laboratory for Conservation and Utilization of Subtropical Agro-Bioresources/Integrative Microbiology Research Centre, South China Agricultural University, Guangzhou, China; ^2^Department of Plant Pathology/Guangdong Province Key Laboratory of Microbial Signals and Disease Control, South China Agricultural University, Guangzhou, China; ^3^State Key Laboratory for Conservation and Utilization of Subtropical Agro-Bioresources, College of Life Science and Technology, Guangxi University, Nanning, China

**Keywords:** filamentous growth, Kpp2, MAP kinase, mating, *Sporisorium scitamineum*, tryptophol

## Abstract

In the phytopathogenic fungus *Sporisorium scitamineum*, sexual mating between two compatible haploid cells and the subsequent formation of dikaryotic hyphae is essential for infection. This process was shown to be commonly regulated by a mitogen-activated protein kinase (MAPK) and a cAMP/PKA signaling pathway in the corn smut fungus *Ustilago maydis* but remains largely unknown in *S. scitamineum*. In this study, we identified a conserved putative MAP kinase Kpp2 in *S. scitamineum* and named it as SsKpp2. The *sskpp2*Δ mutant displayed significant reduction in mating/filamentation, which could be partially restored by addition of cAMP or tryptophol, a quorum-sensing molecule identified in budding yeast. Transcriptional profiling showed that genes governing *S. scitamineum* mating or tryptophol biosynthesis were significantly differentially regulated in the *sskpp2*Δ mutant compared to the WT, under mating condition. Our results demonstrate that the MAP kinase SsKpp2 is required for *S. scitamineum* mating/filamentation likely through regulating the conserved pheromone signal transduction pathway and tryptophol production.

## Introduction

The basidiomycetous fungus *Sporisorium scitamineum* causes sugarcane smut that leads to severe economic losses in the major sugarcane growing areas in China, India, and Brazil. There are three distinct stages in the pathogenic life cycle of *S. scitamineum*, namely the haploid sporidium, the dikaryotic hyphae, and the diploid teliospore stages. The yeast-like and non-pathogenic sporidia are of two opposite mating types, *MAT-1* (+) and *MAT-2* (−). The compatible sporidia fuse to form dikaryotic hypha, which are capable of infecting the host plant. Diploid teliospores form *in planta* by nuclear fusion, and go through a round of meiosis to form four haploid sporidia again (Sundar et al., 2012). Sexual mating and the subsequent filamentous growth (filamentation) is a prerequisite for host cane infection. However, our knowledge on molecular basis of *S. scitamineum* mating/filamentation is very limited.

Mitogen-activated protein kinase (MAPK) signaling pathway is widely conserved in eukaryotic organisms and involved in regulation of cell growth and differentiation, metabolism, stress response including resistance against pathogens in plant or animal hosts (Plotnikov et al., 2011; Meng and Zhang, 2013; Correia et al., 2016). On the other hand, MAPK signaling in fungal pathogens was shown to be closely related to virulence and pathogenicity (Davidson et al., 2003; Roman et al., 2007; Hamel et al., 2012; Jiang et al., 2018). In *Ustilago maydis*, mating/filamentation was regulated by both cAMP/PKA (protein kinase A) signaling pathway and MAPK signaling cascade Kpp4-Fuz7-Kpp2, with partial overlap, and commonly inducing the a locus that encodes the pheromone precursors and receptors through the transcriptional factor Prf1 (Gold et al., 1994; Muller et al., 1999, 2003; Kaffarnik et al., 2003). The MAP kinase Kpp2 is phosphorylated and activated by the upstream kinase Fuz7, and in turn activates its downstream target protein Prf1, the master transcriptional factor for sexual mating in *U. maydis*, to initiate sexual mating in response to pheromone signal (Muller et al., 2003). Kpp2 kinase is conserved in its activation loop (A-loop) motif, especially in the TXY motif containing a Threonine (T) and a Tyrosine (Y) residue that are phosphorylated sites and essential for Kpp2 kinase activity (Chen et al., 2001; Pearson et al., 2001).

Quorum-sensing (QS) plays a role in cell-density based coordinating expression of genes, including virulence genes, in bacterial pathogens (Fuqua et al., 1994; Gray, 1997). Investigation on fungal quorum-sensing molecules (QSMs) initiates recently. So far QSMs have been identified in *Saccharomyces cerevisiae* (Wuster and Babu, 2010), *Candida albicans* (Hornby et al., 2001; Chen and Fink, 2004), *Cryptococcus neoformans* (Albuquerque et al., 2013; Tian et al., 2018), *Aspergillus nidulans* (Williams et al., 2012) and *Penicillium sclerotiorum* (Raina et al., 2010), regulating fungal morphogenesis, pathogenicity, and/or secondary metabolism (Barriuso et al., 2018). Among these fungal QSMs, aromatic alchohols tyrosol, tryptophol, and phenylethanol regulate filamentous growth or pseudohyphae growth in *S. cerevisiae* or *C. albicans* by integrating cell density and nitrogen availability (Chen and Fink, 2006; Wongsuk et al., 2016). These three aromatic alchohols could be produced by Ehrlich pathway (Dickinson et al., 2003; Hazelwood et al., 2008), first step of which is aromatic amino acid deamination catalyzed by aminotransferase Aro8/Aro9, or decarboxylation by DC (amino acid decarboxylase). Following decarboxylation, the aromatic amines could be oxidized by tynA to form the corresponding aldehydes. Particularly, tryptamine is oxidized to indol-3-ylacetaldehyde, which is a direct precursor of both fungal QSM tryptophol and phytohormone auxin/IAA (Indole-3-Acetic Acid; Korasick et al., 2013). Besides as a precursor of tryptophol biosynthesis, tryptophane could also go through kynurenine pathway catalyzed by Bna2/7/4/5/1 and Aro9, to produce an intermediate product quinolinic acid (QA), as a precursor for NAD+ biosynthesis in the budding yeast (Ohashi et al., 2013).

In this study, we identified a conserved *KPP2* gene in *S. scitamineum*, named *SsKPP2*. The *sskpp2*Δ mutants were generated in *MAT-1* and *MAT-2* sporidia background, respectively, and displayed defects in mating/filamentiation and sporidial growth, while no obvious difference in stress response, compared to the wild-type strain. Interestingly, we found that the fungal QSM tryptophol was able to fully restore mating/filamentation between *sskpp2*Δ and wild-type sporidia while not able to restore that between two *sskpp2*Δ sporidia. Using qRT-PCR we assessed transcriptional regulation on the conserved mating/filamentation gene loci a and b locus (Kronstad and Leong, 1990; Bolker et al., 1992; Yan et al., 2016; Lu et al., 2017), and genes encoding critical enzymes in tryptophane metabolism. Our results showed that the a locus genes were significantly down-regulated, and two amine oxidase encoding genes *TYNA1* and *TYNA2* up-regulated, in *sskpp2*Δ mutant compared to the wild-type strain, under mating condition. We further analyzed the deletion mutants of these two *TYNA* genes, *sstyna1*Δ and *sstyna2*Δ mutants, and found that mating/filamentation was reduced between two *sstyna* mutant sporidia. Overall, our study indicating an important function of MAPK signaling pathway in *S. scitamineum* mating/filamentation.

## Results

### Identification of a Conserved MAP Kinase Gene *SsKPP2*

**FIGURE 1:**
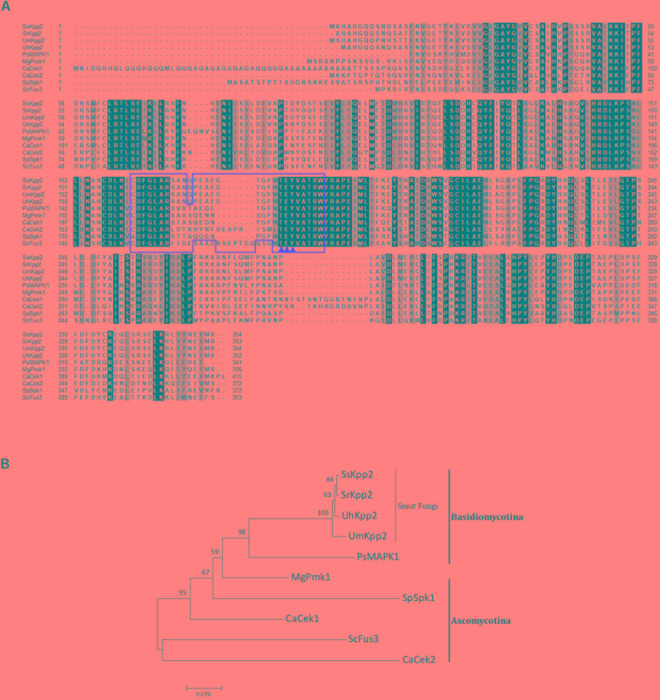
Amino acid sequences arrangement and phylogenetic analysis with SsKpp2 protein and its orthologs. **(A)** Amino acid sequences arrangement and phylogenetic analysis with SsKpp2 protein and its fungal orthologs: *S. reilianum* SrKpp2 (CBQ73711), *U. maydis* UmKpp2 (AAF15528), *U. hordei* UhKpp2 (CCF52019), *Puccinia striiformis* PsMAPK1 (ADL57241), *M. oryzae* MgPmk1 (AAC49521), *C. albicans* CaCek1 (XP_715542) and CaCek2 (AAG43110), *S. pombe* SpSpk1 (NP_594009), and *S. cerevisiae* ScFus3 (NP_009537). The black and gray shadow denote identical and conserved residues, respectively. The red boxes and three red triangles represent STKc_MAPK domains and predicted dual phosphorylation lip sequences, respectively. **(B)** Phylogenetic analysis of Kpp2 othorlogs as listed in **(A)**. The tree is calculated with Neighbor-Joining method (Saitou and Nei, 1987) using MEGA 7 (Kumar et al., 2016). Numbers beside each node indicate a percentage of 1000 bootstrap replications, computed using the Poisson correction method (Zuckerkandl and Pauling, 1965).

Using the *S. cerevisiae* Fus3/Kpp2 protein sequence (NP_009537) to search against the *S. scitamineum* genome available on NCBI website (taxid:49012), via tblastn homology search algorithm^1^, we retrieved a *S. scitamineum* MAP kinase protein Kpp2 (SPSC_04357) of 354 amino acids. Therefore, we named this protein as SsKpp2. Alignment of the SsKpp2 protein with other fungal othorlogs showed a high degree of amino acid conservation, including the conserved TXY motif (amino acid 182–184) in the A-loop (amino acid 162–189, Figure 1A boxed region), which is essential for activation of Kpp2 by phosphorylation (Dhanasekaran and Reddy, 1998). Phylogenetic analysis showed that SsKpp2 is highly conserved, especially within smut fungi (Figure 1B).

The *sskpp2*Δ mutants were generated in wild-type *MAT-1* and *MAT-2* background, respectively, using a modified PEG-mediated protoplast transformation methodology (Yu et al., 2015). The *SsKPP2* open reading frame (ORF) was replaced by the recombinant hygromycin (*HPT*)-resistant selection marker, derived from two PCR amplified truncated but partially overlapped *HPT* fragment flanked by 5′- and 3′- untranslated region (UTR) of the *SsKPP2* ORF (Supplementary Figure S1A). Transformants K17-8 and K17-10 were confirmed as *sskpp2*Δ mutants in *MAT-1* mating-type background, and K18-5 and K18-10 in *MAT-2* background, respectively, by Southern blot (Supplementary Figure S1B). We chose one *sskpp2*Δ mutant in each mating-type background to assess *SsKPP2* gene expression by qPCR, with WT sporidia as control. Our result showed that *SsKPP2* was undetectable in the *sskpp2*Δ mutants (K17-10 and K18-5; Supplementary Figure S1C), further confirming deletion of this gene. In the following, these two mutants were used in various assessment including tolerance to stressful conditions, mating/filamentation, and sporidial (yeast-like) growth.

### Assessment of Stress Tolerance

**FIGURE 2:**
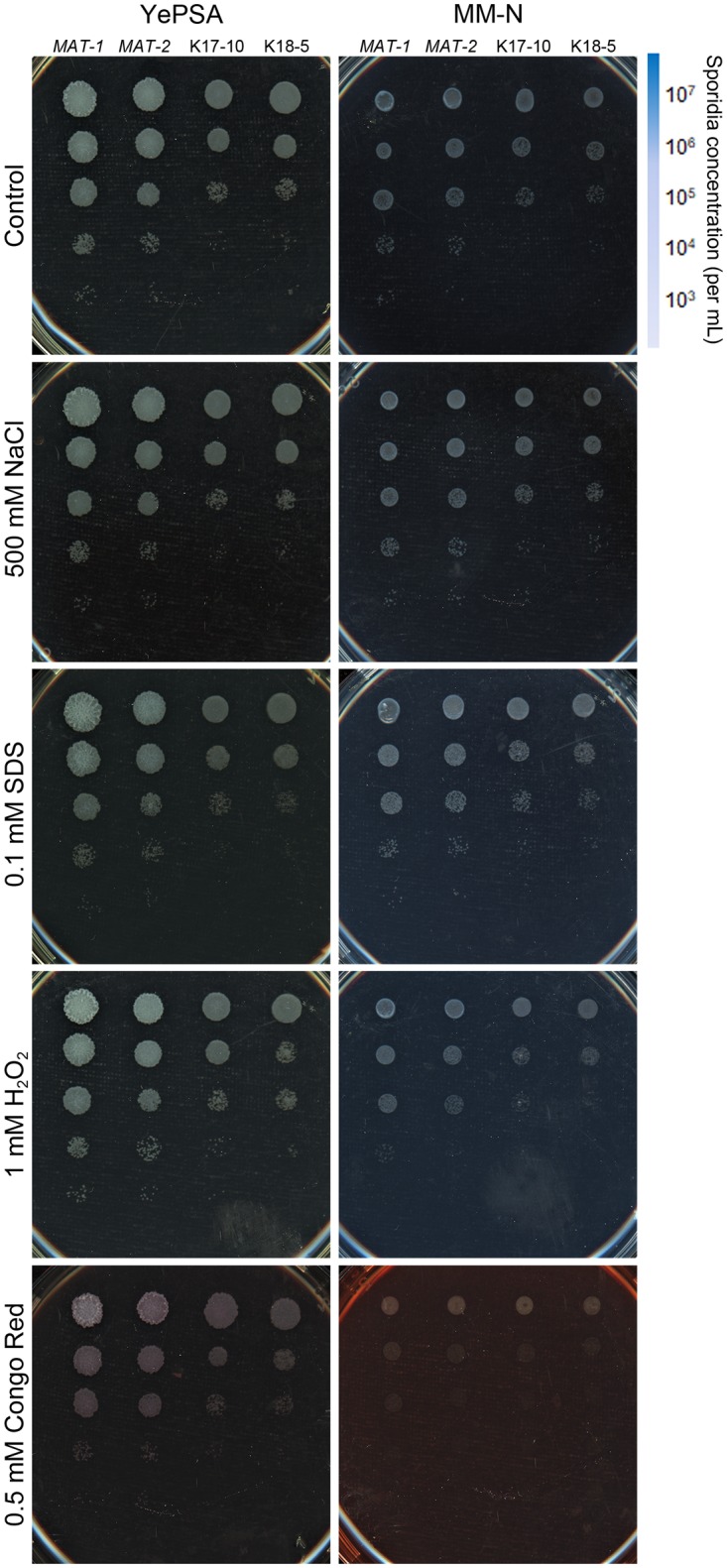
Assessment of tolerance toward stressful conditions. Serially diluted cells of WT or *sskpp2*Δ mutant (Δ) were spotted onto PDA medium supplemented with H_2_O_2_ (1 mM), NaCl (500 mM) or SDS (0.1 mM). Images were taken 72 h post inoculation.

First we examined the tolerance toward various stressful conditions in the wild-type and *sskpp2*Δ sporidia, including cell wall stress (0.1 mM SDS, or 0.5 mM Congo red), hyperosmotic stress (500 mM NaCl), and oxidative stress (1 mM H_2_O_2_). The *sskpp2*Δ sporidia (K17-10 and K18-5) were slightly more sensitive to oxidative stress, or to the cell wall stress caused by Congo red, compared to the WT, when both were cultured on YePSA medium (*MAT-1* and *MAT-2*, Figure 2). Nitrogen starvation (on minimal medium minus nitrogen source, MM-N) made both WT and the mutant sporidia more sensitive to cell wall stressed raised by Congo red, but not by SDS (Figure 2). In summary, we infer that the SsKpp2-mediated MAPK pathway does not seem to be involved in *S. scitamineum* hyperosmotic, oxidative, or cell wall integrity (CWI) stress response.

### SsKPP2 Is Required for *S. scitamineum* Mating/Filamentation

**FIGURE 3:**
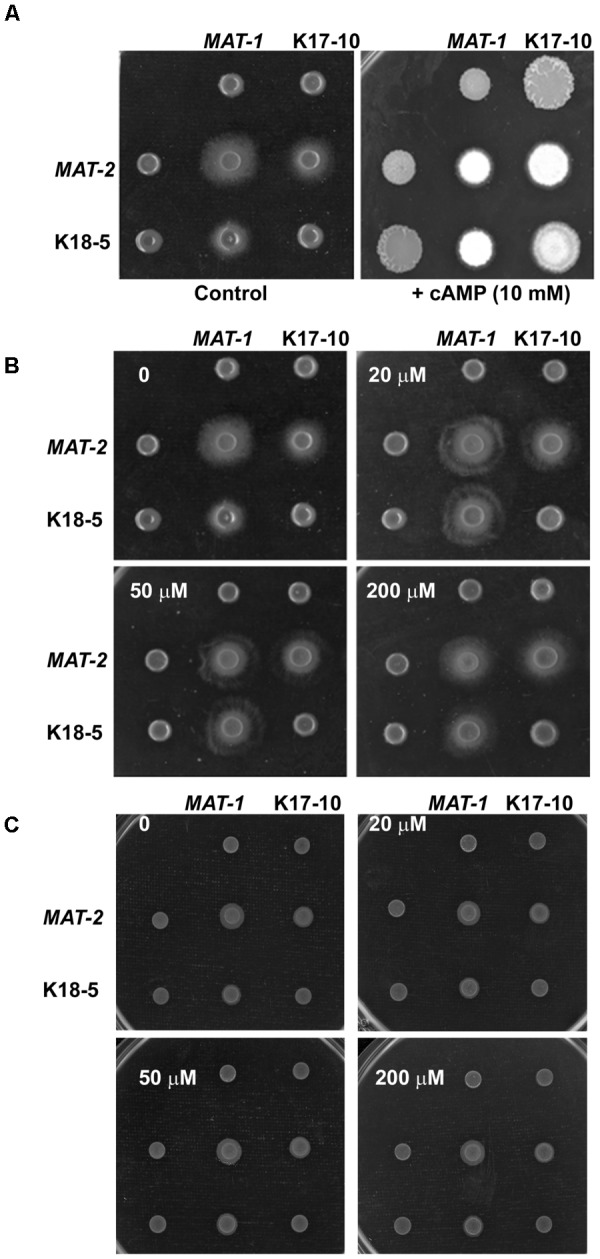
SsKpp2 is essential for *S. scitamineum* mating/filamentation. Mating/filamentation of WT and *sskpp2*Δ mutant was assessed on PDA solid medium, with or without addition of 10 μM cAMP **(A)**, 20–200 μM tryptophol **(B)**, or 20–200 μM tyrosol **(C)**. CK denotes untreated control set. Photographs were taken 3 days post inoculation.

Next we assessed the mating/filamentation of *sskpp2*Δ mutants mixing with WT sporidia of opposite mating-type, or between two *sskpp2*Δ mutants. The *in vitro* culture of mixed WT *MAT-1* and *MAT-2* sporidia served as positive control, which gave rise to dikaryotic hyphae growth and thus had a fluffy appearance of the colonies (Figure 3A). In contrast, mixed cultured WT *MAT-1* with K18-5 (*sskpp2*Δ in *MAT-2* background), or WT *MAT-2* with K17-10 (*sskpp2*Δ in *MAT-1* background) both displayed obviously reduced filamentous and radial growth, while their sporidial colony was comparable to that of WT (Figure 3A). Mating/filamenation was completely blocked in the mating culture of *sskpp2*Δ mutants in two opposite mating-types (Figure 3A). We then tested the effect of cAMP, and two established fungal QSMs tyrosol (Chen et al., 2004) and tryptophol (Wuster and Babu, 2010), respectively, on *sskpp2*Δ filamenation. Addition of 10 mM cAMP could effectively promote filamentation in the mixed culture between WT and *sskpp2*Δ mutants and between two *sskpp2*Δ mutants (Figure 3A), suggesting an overlap between cAMP/PKA and MAPK signaling pathways in regulation of mating/filamentation, similar as reported in *U. maydis* (Kaffarnik et al., 2003). Tryptophol of concentration ranging from 20 to 200 μM could restore filamentous growth in the mixed culture between WT and *sskpp2*Δ mutants, but not in the mating culture between two *sskpp2*Δ mutants (Figure 3B). Tyrosol could not restore *sskpp2*Δ mating/filamentation (Figure 3C).

**FIGURE 4:**
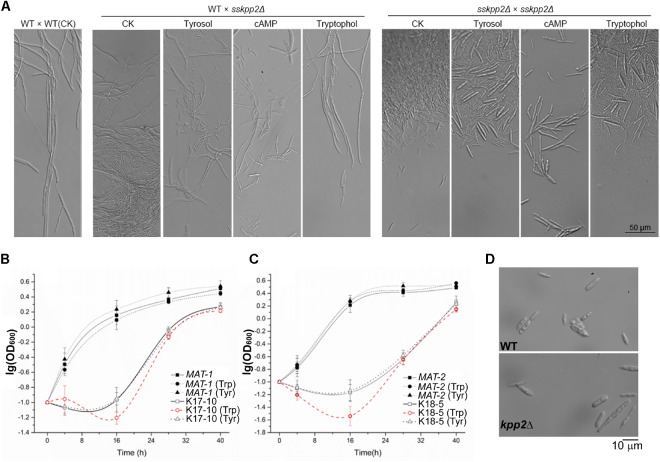
Effect of aromatic chemicals on *S. scitamineum* mating/filamentation or sporidial growth. **(A)** Microscopic imaging of hyphae formed after sexual mating, in WT or *sskpp2*Δ mutant, supplemented with cAMP (10 mM), tyrosol (20 μM) or tryptophol (20 μM), or without supplemented chemicals (CK) as a control. Images were taken 48 h post inoculation. Scale bar = 50 μm. Sporidial growth was assessed in the WT (solid line) or *sskpp2*Δ (dashed line) strain of *MAT-1* **(B)** or *MAT-2* **(C)** mating-type background. *S. scitamineum* sporidia was cultured in liquid YePS medium for 24 h before dilution to reach the concentration of 10^5^ cells per ml. The diluted cells were cultured for a further 40 h in the absence (cube) or presence of tyrosol (20 μM; triangle) or tryptophol (20 μM; circle). The number of cells at each specific time point was acquired by measuring the OD absorption at 600 nm. The growth curve for each culture was prepared by plotting the logarithmic values of OD600 vs. incubation time. Mean ± S.E. are derived from three independent biological repeats, each of which contained three technical repeats. **(D)** Representative microscopic images of wild-type (WT) and the *sskpp2*Δ sporidia. *n* > 40 for each strain examined. Images were taken with Axio Observer Z1 microscope equipped with an sCMOS camera. Scale bar = 10 μm.

Microscopic observation was performed to closely examine the hyphal morphology in the WT and *sskpp2*Δ mutant. Abundant un-mating sporidia were seen in the WT/*sskpp2*Δ mating culture, without any treatment (CK; Figure 4A). Meanwhile, hyphae or pseudohyphae were also observed in this WT/*sskpp2*Δ combination (Figure 4A). In contrast, long and smooth hyphae formed in the WT mating cultured under same condition (Figure 4A). Treatment with tyrosol did not promote hyphae formation in WT/*sskpp2*Δ culture, while addition of or tryptophol did (Figure 4A). However, in *sskpp2*Δ/*sskpp2*Δ culture, even tryptophol could not restore filamentous growth (Figure 4A). Interestingly, addition of cAMP seemed to promote pseudohyphae formation (Figure 4A), which gave rise to the fluffy look of the colonies of WT/*sskpp2*Δ or *sskpp2*Δ/*sskpp2*Δ mating cultures as appeared in Figure 3A. Overall, we found that *SsKPP2* is required for *S. scitamineum* mating/filamentation, which is likely related to tryptophol production.

We further examined the sporidial growth in WT and the *sskpp2*Δ mutant, as well as relationship of tyrosol and tryptophol with *S. scitamineum* cell density. We noticed that the *sskpp2*Δ mutant was obviously delayed in sporidial growth compared to the WT, in both mating-types (Figures 4B,C). Tyrosol could slightly promote such yeast-like growth in WT but not in the *sskpp2*Δ mutants (Figures 4B,C). In contrast, tryptophol could slightly suppress sporidial growth in both WT and the *sskpp2*Δ mutants (Figures 4B,C). This result indicates that the promotion of filametation by tryptophol (Figure 3B) was not due to change in growth rate of haploid sporidia. In summary, we found that SsKpp2 is also involved in regulation on *S. scitamineum* cell growth (division).

Furthermore, we compared the spordial morphology of the wild-type (*MAT-1* and *MAT-2*) and the *sskpp2*Δ mutants (K17-10 and K18-5) by microscopy. As shown in Figure 4D, the *sskpp2*Δ sporidia appeared cigar-shape and identical to those of the wild-type strains. We measured the sporidial size and found that the length of the *sskpp2*Δ sporidia is significantly (*p* < 0.001) longer than that of WT, while no difference in the width of WT and *sskpp2*Δ sporidia (Table 1). These results suggest that SsKpp2 is not required for sporidial morphogenesis.

**Table 1:** Measurement of sporidial size of WT (*MAT-1* and *MAT-2*) and *sskpp2*Δ mutants (K17-10 and K18-5).

Strain	Size (length × width)^∗^
*MAT-1*	10.50 ± 1.39^A^ × 3.12 ± 0.46^C^ μm
*MAT-2*	13.31 ± 2.22^B^ × 3.35 ± 0.55^C^ μm
K17-10	11.99 ± 2.29^A^ × 3.31 ± 0.42^C^ μm
K18-5	14.85 ± 1.87^B^ × 3.31 ± 0.56^C^ μm

*^∗^numbers represent Average ± S.E., derived from three independent repeats, with >40 sporidia of each strain measured for each repeat. Different capitalized letters represent significant difference statistically.*

### *SsKPP2* Is Involved in Transcriptional Regulation of Mating/Filamentation Genes or Tryptophol Biosynthesis During *S. scitamineum* Mating/Filamentation

**FIGURE 5:**
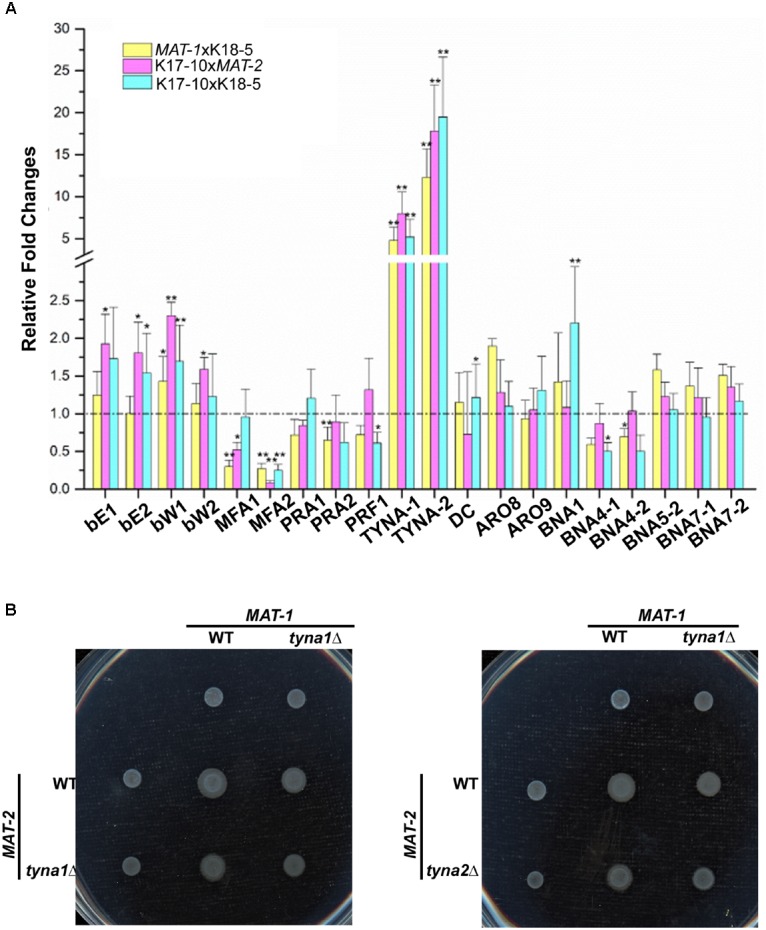
Identification of potential target genes of Sskpp2-mediated MAPK signaling pathway. **(A)** qRT-PCR analysis with selected genes in the *sskpp2*Δ mutant in comparison to WT, under mating condition. Relative gene expression fold change was calculated with –ΔΔCt method (Livak and Schmittgen, 2001) with *ACTIN* as internal control. Mean ± S.E. are derived from two independent biological repeats, each of which contained three replica. ^∗^ and ^∗∗^ denote significant difference with *p* < 0.05 and *p* < 0.01, respectively. **(B)** Mating/filamentation of WT, *sstyna1*Δ and *sstyna2*Δ mutants was assessed on PDA solid medium. Photographs were taken 3 days post inoculation.

To get a better understanding of the regulatory mechanism on governing *S. scitamineum* mating/filamentation via SsKpp2-mediated MAPK signaling pathway, we performed a qRT-PCR analysis with genes related to fungal mating/filamentation and tryptophane metabolism (Supplementary Table S1). Our results showed that indeed the a locus genes *MFA1* and *MFA2* were significantly down-regulated, while the b locus gene *bE1*, *bE2*,*bW1*, and *bW1* up-regulated (Figure 5A). The mating/filamentation master regulator, Prf1, was also down-regulated especially in *sskpp2*Δ/*sskpp2*Δ culture (Figure 5A). Among the genes regulating tryptophol production, only two *TYNA* genes (*SPSP_0335* and *SPSC_0449*) were highly up-regulated (Figure 5A). The tynA enzyme catalyzes conversion from tryptamine to indol-3-ylacetaldehyde, the direct precursor for the production of tryptophol in Ehrlich pathway (Hazelwood et al., 2008) or the phytohormone IAA (Korasick et al., 2013). We inferred that up-regulation of *TYNA* genes in the *sskpp2*Δ mutant may correlate to its defects in mating/filamentation.

To verify such hypothesis, we generated *sstyna1*Δ and *sstyna2*Δ mutant, respectively (Supplementary Figures S2A–C) and tested their mating/filamentation. As both of *sstyna1*Δ and *sstyna2*Δ mutant could mating with WT and form filaments only slightly reduced compared to WT mating (*MAT-1*/*MAT-2*), mating/filamentation in two mutant sporidia, *sstyna1*Δ/*sstyna1*Δ or *sstyna1*Δ/*sstyna2*Δ, seemed further reduced (Figure 5B). Overall, we found that SsKpp2 was required for *S. scitamineum* mating/filamentation, likely via regulation on pheromone signal transduction and tryptohol biosynthesis.

## Discussion

Mitogen-activated protein kinase is protein kinase highly conserved in eukaryotic organisms as signal transducers (Kultz and Burg, 1998; Roman et al., 2007). In this study, we identified a *S. scitamineum* ortholog of *KPP2* gene, that encodes a MAP kinase most closely related to other fungal MAP kinases including Pmk1 of *M. oryzae*, Cek1 and Cek2 of *C. albicans*, Spk1 of *S. pombe*, Fus3p of *S. cerevisiae*, and in several other basidiomycetous fungi especially smut fungi. The phylogenetic analysis showed that SsKpp2 protein is highly conserved among smut fungi, and along with ascomycetes it is conserved in a typical STKc_MAPKs domain (A-loop) with the dual phosphorylation lip sequence TXY is present in all the fungal Kpp2s. Functions in fungal development and pathogenesis was reported with aforementioned Kpp2 othorlogs. For example, *M. oryzae* Pmk1 for appressoria formation and plant cell-to-cell invation (Zhao et al., 2005; Sakulkoo et al., 2018). *C. albicans* Cek1 is involved in morphogenesis and hyphal formation (Csank et al., 1998) while Cek2 participates in sexual mating (Chen et al., 2002), and both of them required for dimorphic switch, virulence, and cell wall integrity and partially functionally redundant (Correia et al., 2016). *U. maydis* Kpp2 regulates sexual mating/filamentation and host infection (Muller et al., 1999). However, Kpp2 kinase has not been identified or functionally characterized in *S. scitamineum*.

In this current study, we generated and characterized the *sskpp2*Δ mutants in both mating-types in *S. scitamineum* and the result showed that deletion of *SsKPP2* does not change hyperosmotic, oxidative, or CWI stress response in the sporidial growth stage. However, mating/filamentation was significantly reduced in WT/*sskpp2*Δ culture and completely blocked in *sskpp2*Δ/*sskpp2*Δ culture, indicating that SsKpp2 function is essential for *S. scitamineum* mating/filamentation. This is consistent with what has been reported in budding yeast, that the MAPK cascade Hog1 is mainly involved in hyperosmotic stress response, and Mpk1/Slt2 cascade in CWI, while Fus3 and Kss1 cascades responsible for pheromone response and/or filamentation (Chen and Thorner, 2007; Waltermann and Klipp, 2010). SsKpp2 function also seems conserved with its *U. maydis* ortholog (Muller et al., 2003). Transcriptional profiling confirmed that expression of *PRF1* orthologous gene, encoding the master regulator of fungal mating/filamentation in response to pheromone signal, was reduced in mating cultures containing *sskpp2*Δ, so as to the a locus genes, which depend on Prf1 for transcriptional activation (Hartmann et al., 1996). However, at present it remains to be confirmed whether SsKpp2 directly phosphorylates Prf1 for its activation, before we could further investigate the mechanism underlying SsKpp2 regulation on *S. scitamineum* mating/filamentation. Overall we infer that the MAPK signaling pathway mediated by SsKpp2 may regulate *S. scitamineum* mating/filamentation through Prf1 activation of pheromone response genes, similar as that in *U. maydis*.

Two aromatic alcohols, tyrosol and tryptophol, have been reported as fungal QSMs in *C. albicans* in *S. cerevisiae*, respectively, in promoting filamentation integrating cell density and/or nitrogen availability (Chen et al., 2004; Wongsuk et al., 2016). Feedback regulation of tryptophol production is mediated by cAMP/PKA signaling pathway in *S. cerevisiae* while not in *C. albicans* (Chen and Fink, 2006). It was not reported on any signaling pathway regulating tyrosol production in *C. albicans* dimorphic switch, but a two-component system and MAPK (Cek1) signaling regulation on production of another fungal QSM farnesol, was reported (Kruppa et al., 2004; Roman et al., 2009). Although the MAPK signal cascade regulation on mating/filamentation in response to pheromone signals were elucidated in *U. maydis* (Muller et al., 2003), its connection to fungal quorum sensing (if any) has not been reported in this smut fungus, neither was any fungal QSM(s) identified in smut fungi.

Interestingly, in our study we found that addition of tryptophol but not tyrosol could restored mating/filamentation in WT/*sskpp2*Δ culture, and a transcriptional regulation of *TYNA* genes involved in tryptophol biosynthesis was indicated by qRT-PCR analysis as well. Mating between two *TYNA* genes deletion mutants resulted in reduced mating/filamentation, suggesting that tryptophol (or other intermediate product in tryptophane catabolism) may likely play a role in *S. scitamineum* mating/filamentation and under MAPK signal regulation. We notice that mating/filamentation in sporidia combination of *sstyna1*Δ/*sstyna1*Δ or *sstyna1*Δ/*sstyna2*Δ seemed further reduced compared to WT/*sstyna1*Δ or WT/*sstyna2*Δ combination, indicating that there may be a functional redundancy between these two copies of *TYNA* genes in *S. scitamineum*. Such integrated regulation of pheromone signal and potential quorum-sensing signal via MAP kinase function has not been reported in other fungi, although it is pending verification whether tryptophol actually acts as a fungal quorum-sensing molecule in *S. scitamineum.*

In summary, our study provides evidence that *S. scitamineum* MAP kinase SsKpp2 is required for proper mating/filamentation, likely via regulation on fungal pheromone response and tryptophol biosynthesis.

## Materials and Methods

### Growth Conditions and Fungal Strains Used in This Study

Teliospores of sugarcane smut collected from the fields in Guangdong province of China (21°12′ 36″ N; 101°10′ 12″ E) by Yan et al. (2016) was maintained in Z. Jiang’s lab, and the *MAT-1* or *MAT-2* haploid sporidia isolated from such teliospores were used in this study. The culture medium used in this study include YePSA medium (yeast extract 1%, peptone 2%, sugar 2%, agar 2%), YePS liquid medium (yeast extraction 1%, peptone 2%, sugar 2%, pH7.0), YePS soft medium (yeast extract 1%, peptone 2%, sugar 2%, agar 0.65%), YePSS medium (yeast extract 1%, peptone 2%, sugar 2%, D-sorbitol 18.17%, agar 2%), PDA (Beijing dingguo, HB0233-12) medium (2% agar, PH7.5), and MM-N medium (minimal medium minus nitrogen source, following the established recipe (Sherman, 2002) without addition of nitrogen source). For stress tolerance assessment, WT or *sskpp2*Δ mutant sporidia of serial diluted concentration from 10^7^ to 10^3^ per mL were inoculated on YePSA or MM-N medium, and allowed to grow in dark at 28°C incubator for 3 days before photographing. For mating/filamentation assay, the equal volume of wild-type or deletion mutant’s haploid sporidia of opposite mating-types were mixed and plated on the solid medium, and kept in dark at 28°C incubator for 2–3 days before photographing. For growth assay, sporidia of *S. scitamineum* wild-type or *sskpp2*Δ mutants were cultured in 5 mL of YePS liquid medium at 28°C, with shaking at 200 rpm for 24 h. An aliquot of such cultured sporidia were then diluted to fresh YePS liquid medium, adjusting to cell density of 10^5^ cells per ml, and cultured for another 40 h under the same condition. Measurement of O.D.600 with spectrophotometer (Thermo, NanoDrop 2000C) was performed hourly to monitor the yeast-like (budding) growth of wild-type or mutant strains, with or without addition of chemical reagents as described in the Results.

### Chemical Compounds Used in This Study

Tyrosol (Sigma-Aldrich, 188255); tryptophol (Sigema-Aldrich, V900672); cAMP (Sigma-Aldrich, A9501).

### Generation of Deletion Constructs

The primers used for generating *sskpp2*Δ, *tyna-1*Δ, and *tyna-2*Δ mutants were listed in Supplementary Table S2. Targeted gene deletion follows the strategy described (Chung et al., 2002; Yang and Chung, 2012), by PCR amplification of two fragments flanking the targeted gene, each of which fused with half partial-overlapping *HPT* (Hyg^r^ gene) sequence. The flanking DNA (1kb 5′- and 3′-) fragments were amplified using wild-type *S. scitamineum* genomic DNA as template, and the *HPT* gene with plasmid pEX2 (Yan et al., 2016) as template.

### Nucleic Acid Related Manipulation

Fungal genomic DNA was extracted using a HP Fungal DNA Kit (Omega,D3195-01). PCR amplification was performed using Phusion High-Fidelity DNA Polymerase (Thermo Scientific,lot:00528748). DNA fragment elution was performed using Gel Extraction Kit (Omega,D2500-02) and/or Cycle Pure Kit (Omega, D6492-02). In Southern blot assay, restriction enzymes used for digestion of genomic DNA were from NEB [NewEnglandBiolabs (Beijing) Ltd.]. Labeling and Detection Starter Kit I (Roche, 11745832910) was used for labeling of PCR amplified fragments as probe. Amersham Hybond TM-N+ (GE Healthcare, RFN303B) membrane was used for blotting. NBT/BCIP Stock Solution (Roche,11681451001) was used for probed band detection. For total RNA extraction, Qiagen RNeasy Plant Mini kit (74104) was used. Ambion^®^ TURBO DNA-free^TM^ kit (Invitrogen, AM1907) was used for removing contaminating DNA from RNA preparations. TransScript^®^ First-Strand cDNA Synthesis Super Mix (Transgen, AT301-02) was used for cDNA systhesis. For real-time qPCR we used PowerUp^TM^ SYBR^®^ Green Master Mix (Applied Biosystems, A25742) and the reaction was run on QuantStudio 6 Flex Real-Time PCR System (Thermo Fisher Scientific). The primers used for qRT-PCR analysis were listed in Supplementary Table S3.

### PEG (Polyethylene Glycol) – Mediated Protoplast Transformation

Polyethylene glycol-mediated protoplast transformation follows the established protocol (Yu et al., 2015) with modification: Wild-type *MAT*-1 or *MAT-2* sporidia was incubated with the lysing enzyme (Sigma L1412) of 4 mg/mL, dissolved in SCS solution (20 mM trisodium citrate and 1 M D-sorbitol, pH 5.8), at 28°C for 30 min for enzyme digestion of fungal cell wall. 40% PEG (Sigema-Aldrich,202444) solution was prepared in 10 mL STC solution (10 mM Tris–HCl, pH 7.5; 1 M D-sorbitol and 100 mM CaCl2). 1∼5 μg of the PCR amplified fragments were mixed with 1 μl heparin solution (15 mg/ml; Beijing dingguo,DH157) and the protoplasts, and incubated with 40% PEG solution on ice for 10 min. The protoplasts were regenerated on the 3-layer regeneration medium composed of top layer of YePS soft medium plus two lays of YePSS medium, with only the bottom YePSS layer containing 400 μg/ml hygromycin B (Calbiochem,CAS:53-84-9) for primary screening of transformants based on antibiotic resistance.

### Microscopy

Images were taken with Axio Observer Z1 microscope (Zeiss, Jena, Germany) equipped with an sCMOS camera (PCO Edge, Kelheim, Germany).

### Statistic Analysis

Data were expressed as mean ± standard error (SE). Differences among different treatments were analyzed using Student’s *t*-test formula in Microsoft Excel.

## Author Contributions

YD, BC, and ZJ conceived and designed the experiments. BZ, CC, YW, SS, XZ, and SL performed the experiments. YD, CC, BC, and ZJ analyzed the data and wrote the manuscript.

## Conflict of Interest Statement

The authors declare that the research was conducted in the absence of any commercial or financial relationships that could be construed as a potential conflict of interest.

## Footnotes

1 https://blast.ncbi.nlm.nih.gov/Blast.cgi?PROGRAM=tblastn&PAGE_TYPE=BlastSearch&LINK_LOC=blasthome

## References

[B1] Albuquerque P. Nicola A. M. Nieves E. Paes H. C. Williamson P. R. Silva-Pereira I. (2013). Quorum sensing-mediated, cell density-dependent regulation of growth and virulence in *Cryptococcus neoformans*. *mBio* 5:e00986-13. 10.1128/mBio.00986-13 24381301 PMC3884061

[B2] Barriuso J. Hogan D. A. Keshavarz T. Martínez M. J. (2018). Role of quorum sensing and chemical communication in fungal biotechnology and pathogenesis. *FEMS Microbiol. Rev.* 42 627–638. 10.1093/femsre/fuy022 29788231 PMC6098223

[B3] Bolker M. Urban M. Kahmann R. (1992). The a mating type locus of *U. maydis* specifies cell signaling components. *Cell* 68 441–450. 10.1016/0092-8674(92)90182-C 1310895

[B4] Chen H. Fink G. R. (2004). Tyrosol is a quorum-sensing molecule in *Candida albicans*. *Proc. Natl. Acad. Sci. U.S.A.* 101 5048–5052. 10.1073/pnas.0401416101 15051880 PMC387371

[B5] Chen H. Fink G. R. (2006). Feedback control of morphogenesis in fungi by aromatic alcohols. *Genes Dev.* 20 1150–1161. 10.1101/gad.1411806 16618799 PMC1472474

[B6] Chen H. Fujita M. Feng Q. Clardy J. Fink G. R. (2004). Tyrosol is a quorum-sensing molecule in *Candida albicans*. *Proc. Natl. Acad. Sci. U.S.A.* 101 5048–5052. 10.1073/pnas.0401416101 15051880 PMC387371

[B7] Chen J. Chen J. Lane S. Liu H. (2002). A conserved mitogen-activated protein kinase pathway is required for mating in *Candida albicans*. *Mol. Microbiol.* 46 1335–1344. 10.1046/j.1365-2958.2002.03249.x 12453219

[B8] Chen R. E. Thorner J. (2007). Function and regulation in MAPK signaling pathways: lessons learned from the yeast *Saccharomyces cerevisiae*. *Biochim. Biophys. Acta* 1773 1311–1340. 10.1016/j.bbamcr.2007.05.003 17604854 PMC2031910

[B9] Chen Z. Gibson T. B. Robinson F. Silvestro L. Pearson G. Xu B. (2001). MAP kinases. *Chem. Rev.* 101 2449–2476. 10.1021/cr000241p 11749383

[B10] Chung K. R. Shilts T. Li W. Timmer L. W. (2002). Engineering a genetic transformation system for *Colletotrichum acutatum*, the causal fungus of lime anthracnose and postbloom fruit drop of citrus. *FEMS Microbiol. Lett.* 213 33–39. 10.1111/j.1574-6968.2002.tb11282.x 12127485

[B11] Correia I. Roman E. Prieto D. Eisman B. Pla J. (2016). Complementary roles of the Cek1 and Cek2 MAP kinases in *Candida albicans* cell-wall biogenesis. *Future Microbiol.* 11 51–67. 10.2217/fmb.15.127 26682470

[B12] Csank C. Schroppel K. Leberer E. Harcus D. Mohamed O. Meloche S. (1998). Roles of the *Candida albicans* mitogen-activated protein kinase homolog. Cek1p, in hyphal development and systemic candidiasis. *Infect. Immun.* 66 2713–2721. 9596738 10.1128/iai.66.6.2713-2721.1998 PMC108260

[B13] Davidson R. C. Nichols C. B. Cox G. M. Perfect J. R. Heitman J. (2003). A MAP kinase cascade composed of cell type specific and non-specific elements controls mating and differentiation of the fungal pathogen *Cryptococcus neoformans*. *Mol. Microbiol.* 49 469–485. 10.1046/j.1365-2958.2003.03563.x 12828643

[B14] Dhanasekaran N. Reddy E. (1998). Signaling by dual specificity kinases. *Oncogene* 17 1447–1455. 10.1038/sj.onc.1202251 9779990

[B15] Dickinson J. R. Salgado L. E. Hewlins M. J. (2003). The catabolism of amino acids to long chain and complex alcohols in *Saccharomyces cerevisiae*. *J. Biol. Chem.* 278 8028–8034. 10.1074/jbc.M211914200 12499363

[B16] Fuqua W. C. Winans S. C. Greenberg E. P. (1994). Quorum sensing in bacteria: the LuxR-LuxI family of cell density-responsive transcriptional regulators. *J. Bacteriol.* 176 269–275. 10.1128/jb.176.2.269-275.1994 8288518 PMC205046

[B17] Gold S. Duncan G. Barrett K. Kronstad J. (1994). cAMP regulates morphogenesis in the fungal pathogen *Ustilago maydis*. *Genes Dev.* 8 2805–2816. 10.1101/gad.8.23.2805 7995519

[B18] Gray K. M. (1997). Intercellular communication and group behavior in bacteria. *Trends Microbiol.* 5 184–188. 10.1016/S0966-842X(97)01002-0 9160506

[B19] Hamel L. P. Nicole M. C. Duplessis S. Ellis B. E. (2012). Mitogen-activated protein kinase signaling in plant-interacting fungi: distinct messages from conserved messengers. *Plant Cell* 24 1327–1351. 10.1105/tpc.112.096156 22517321 PMC3398478

[B20] Hartmann H. A. Kahmann R. Bolker M. (1996). The pheromone response factor coordinates filamentous growth and pathogenicity in *Ustilago maydis*. *EMBO J.* 15 1632–1641. 10.1002/j.1460-2075.1996.tb00508.x 8612587 PMC450074

[B21] Hazelwood L. A. Daran J. M. van Maris A. J. Pronk J. T. Dickinson J. R. (2008). The Ehrlich pathway for fusel alcohol production: a century of research on *Saccharomyces cerevisiae* metabolism. *Appl. Environ. Microbiol.* 74 2259–2266. 10.1128/aem.02625 18281432 PMC2293160

[B22] Hornby J. M. Jensen E. C. Lisec A. D. Tasto J. J. Jahnke B. Shoemaker R. (2001). Quorum sensing in the dimorphic fungus *Candida albicans* is mediated by farnesol. *Appl. Environ. Microbiol.* 67 2982–2992. 10.1128/AEM.67.7.2982-2992.2001 11425711 PMC92970

[B23] Jiang C. Zhang X. Liu H. Xu J. R. (2018). Mitogen-activated protein kinase signaling in plant pathogenic fungi. *PLoS Pathog.* 14:e1006875. 10.1371/journal.ppat.1006875 29543901 PMC5854419

[B24] Kaffarnik F. Muller P. Leibundgut M. Kahmann R. Feldbrugge M. (2003). PKA and MAPK phosphorylation of Prf1 allows promoter discrimination in *Ustilago maydis*. *EMBO J.* 22 5817–5826. 10.1093/emboj/cdg554 14592979 PMC275411

[B25] Korasick D. A. Enders T. A. Strader L. C. (2013). Auxin biosynthesis and storage forms. *J. Exp. Bot.* 64 2541–2555. 10.1093/jxb/ert080 23580748 PMC3695655

[B26] Kronstad J. W. Leong S. A. (1990). The b mating-type locus of *Ustilago maydis* contains variable and constant regions. *Genes Dev.* 4 1384–1395. 10.1101/gad.4.8.1384 2227416

[B27] Kruppa M. Krom B. P. Chauhan N. Bambach A. V. Cihlar R. L. Calderone R. A. (2004). The two-component signal transduction protein Chk1p regulates quorum sensing in *Candida albicans*. *Eukaryot. Cell* 3 1062–1065. 10.1128/ec.3.4.1062-1065.2004 15302838 PMC500889

[B28] Kultz D. Burg M. (1998). Evolution of osmotic stress signaling via MAP kinase cascades. *J. Exp. Biol.* 201(Pt 22), 3015–3021. 9787121 10.1242/jeb.201.22.3015

[B29] Kumar S. Stecher G. Tamura K. (2016). MEGA7: molecular evolutionary genetics analysis version 7.0 for bigger datasets. *Mol. Biol. Evol.* 33 1870–1874. 10.1093/molbev/msw054 27004904 PMC8210823

[B30] Livak K. J. Schmittgen T. D. (2001). Analysis of relative gene expression data using real-time quantitative PCR and the 2(-Delta Delta C(T)) Method. *Methods* 25 402–408. 10.1006/meth.2001.1262 11846609

[B31] Lu S. Shen X. Chen B. (2017). Development of an efficient vector system for gene knock-out and near in-cis gene complementation in the sugarcane smut fungus. *Sci. Rep.* 7:3113. 10.1038/s41598-017-03233-7 28596577 PMC5465213

[B32] Meng X. Zhang S. (2013). MAPK cascades in plant disease resistance signaling. *Annu. Rev. Phytopathol.* 51 245–266. 10.1146/annurev-phyto-082712-102314 23663002

[B33] Muller P. Aichinger C. Feldbrugge M. Kahmann R. (1999). The MAP kinase kpp2 regulates mating and pathogenic development in *Ustilago maydis*. *Mol. Microbiol.* 34 1007–1017. 10.1046/j.1365-2958.1999.01661.x 10594825

[B34] Muller P. Weinzierl G. Brachmann A. Feldbrugge M. Kahmann R. (2003). Mating and pathogenic development of the Smut fungus *Ustilago maydis* are regulated by one mitogen-activated protein kinase cascade. *Eukaryot. Cell* 2 1187–1199. 10.1128/EC.2.6.1187-1199.2003 14665454 PMC326639

[B35] Ohashi K. Kawai S. Murata K. (2013). Secretion of quinolinic acid, an intermediate in the kynurenine pathway, for utilization in NAD + biosynthesis in the yeast *Saccharomyces cerevisiae*. *Eukaryot. Cell* 12 648–653. 10.1128/EC.00339-12 23457190 PMC3647768

[B36] Pearson G. Robinson F. Beers Gibson T. Xu B. E. Karandikar M. Berman K. (2001). Mitogen-activated protein (MAP) kinase pathways: regulation and physiological functions. *Endocr. Rev.* 22 153–183. 10.1210/edrv.22.2.0428 11294822

[B37] Plotnikov A. Zehorai E. Procaccia S. Seger R. (2011). The MAPK cascades: signaling components, nuclear roles and mechanisms of nuclear translocation. *Biochim. Biophys. Acta* 1813 1619–1633. 10.1016/j.bbamcr.2010.12.012 21167873

[B38] Raina S. Odell M. Keshavarz T. (2010). Quorum sensing as a method for improving sclerotiorin production in *Penicillium sclerotiorum.* *J. Biotechnol.* 148 91–98. 10.1016/j.jbiotec.2010.04.009 20438771

[B39] Roman E. Alonso-Monge R. Gong Q. Li D. Calderone R. Pla J. (2009). The Cek1 MAPK is a short-lived protein regulated by quorum sensing in the fungal pathogen *Candida albicans*. *FEMS Yeast Res.* 9 942–955. 10.1111/j.1567-1364.2009.00545.x 19656200

[B40] Roman E. Arana D. M. Nombela C. Alonso-Monge R. Pla J. (2007). MAP kinase pathways as regulators of fungal virulence. *Trends Microbiol.* 15 181–190. 10.1016/j.tim.2007.02.001 17321137

[B41] Saitou N. Nei M. (1987). The neighbor-joining method: a new method for reconstructing phylogenetic trees. *Mol. Biol. Evol.* 4 406–425. 10.1093/oxfordjournals.molbev.a040454 3447015

[B42] Sakulkoo W. Oses-Ruiz M. Oliveira Garcia E. Soanes D. M. Littlejohn G. R. Hacker C. (2018). A single fungal MAP kinase controls plant cell-to-cell invasion by the rice blast fungus. *Science* 359 1399–1403. 10.1126/science.aaq0892 29567712

[B43] Sherman F. (2002). Getting started with yeast. *Methods Enzymol.* 350 3–41. 10.1016/S0076-6879(02)50954-X 12073320

[B44] Sundar A. R. Barnabas E. L. Malathi P. Viswanathan R. (2012). *A Mini-Review on Smut Disease of Sugarcane Caused by Sporisorium scitamineum.* London: InTech Open.

[B45] Tian X. He G. J. Hu P. Chen L. Tao C. Cui Y. L. (2018). *Cryptococcus neoformans* sexual reproduction is controlled by a quorum sensing peptide. *Nat. Microbiol.* 3 698–707. 10.1038/s41564-018-0160-4 29784977 PMC12813696

[B46] Waltermann C. Klipp E. (2010). Signal integration in budding yeast. *Biochem. Soc. Trans.* 38 1257–1264. 10.1042/BST0381257 20863295

[B47] Williams H. E. Steele J. C. Clements M. O. Keshavarz T. (2012). γ-Heptalactone is an endogenously produced quorum-sensing molecule regulating growth and secondary metabolite production by *Aspergillus nidulans*. *Appl. Microbiol. Biotechnol.* 96 773–781. 10.1007/s00253-012-4065-5 22543352

[B48] Wongsuk T. Pumeesat P. Luplertlop N. (2016). Fungal quorum sensing molecules: role in fungal morphogenesis and pathogenicity. *J. Basic Microbiol.* 56 440–447. 10.1002/jobm.201500759 26972663

[B49] Wuster A. Babu M. M. (2010). Transcriptional control of the quorum sensing response in yeast. *Mol. Biosyst.* 6 134–141. 10.1039/b913579k 20024075

[B50] Yan M. Zhu G. Lin S. Xian X. Chang C. Xi P. (2016). The mating-type locus b of the sugarcane smut *Sporisorium scitamineum* is essential for mating, filamentous growth and pathogenicity. *Fungal Genet. Biol.* 86 1–8. 10.1016/j.fgb.2015.11.005 26563415

[B51] Yang S. L. Chung K. R. (2012). The NADPH oxidase-mediated production of hydrogen peroxide (H_2_O_2_) and resistance to oxidative stress in the necrotrophic pathogen *Alternaria alternata* of citrus. *Mol. Plant Pathol.* 13 900–914. 10.1111/j.1364-3703.2012.00799.x 22435666 PMC6638813

[B52] Yu J. Zhang Y. Cui H. Hu P. Yu X. Ye Z. (2015). An efficient genetic manipulation protocol for *Ustilago esculenta*. *FEMS Microbiol. Lett.* 362:fnv087. 10.1093/femsle/fnv087 26038251

[B53] Zhao X. Kim Y. Park G. Xu J. R. (2005). A mitogen-activated protein kinase cascade regulating infection-related morphogenesis in *Magnaporthe grisea*. *Plant Cell* 17 1317–1329. 10.1105/tpc.104.029116 15749760 PMC1088005

[B54] Zuckerkandl E. Pauling L. (1965). Evolutionary divergence and convergence in proteins. *Evol. Genes Proteins* 97 97–166. 10.1016/B978-1-4832-2734-4.50017-6

