# Supplementary material for: The MAP Kinase SsKpp2 Is Required for Mating/Filamentation in Sporisorium scitamineum
Source: Front Microbiol. 2018 Oct 26;9:2555. doi: 10.3389/fmicb.2018.02555 (PMC6212578; doi:10.3389/fmicb.2018.02555)
Supplement: Supplementary file 1 [file Data_Sheet_1.pdf]

Supplementary Information

The MAP kinase SsKpp2 is required for mating/filamentation in *Sporisorium scitamineum*

Yi Zhen Deng[1], Bin Zhang, Changqing Chang, Yixu Wang, Shan Lu, Sun Shuquan, Xiaomeng Zhang, Baoshan Chen, Zide Jiang[1]

1. To whom correspondence should be addressed. Email: zdjiang@scau.edu.cn (Z Jiang) or dengyz@scau.edu.cn (YZ Deng).

This PDF file includes

Figures and Table

Figure S1 Generation and verification of *sskpp2*Δ mutants. (A) schematic representation of *S. scitamineum SsKPP2* locus (*SPSC_04357*) drawn to scale, wherein, open arrowed bars represent coding region (and its orientation) and dashed lines denote the homologous region constructed flanking the hygromycin-resistance cassette (*HPT*). The solid line labelled with "probe" denotes the location and length of the DNA fragment used as a probe to verify the transformants by Southern blot as shown in (B). Distribution of restriction enzyme *Xho*I in the genomic region outside the homologous regions (solid line), or in *HPT* gene, was indicated. Scale bar = 1 kb. (B) Southern blot analysis to confirm *SsKPP2* deletion. Genomic DNA from wild type (WT) or transformants from two mating-type background (*MAT-1* and *MAT-2*) was digested with *Xho*I, and then probed with the probe fragment as indicated in (A). Detection of the *HPT* gene fragment of expected size (2 kb) was dignostic as deletion mutants. (C) The *sskpp2*Δ mutants (K17-10 and K18-5) in two mating-type backgrounds (*MAT-1* and *MAT-2*) were confirmed by Semiquantitative RT-PCR using gene-specific primers of the *SsKPP2*. Relative gene expression level was calculated with -ΔΔCt method (Livak & Schmittgen, 2001) with *ACTIN* as internal control. All primers were listed in Table S2 or Table S3.

Figure S1

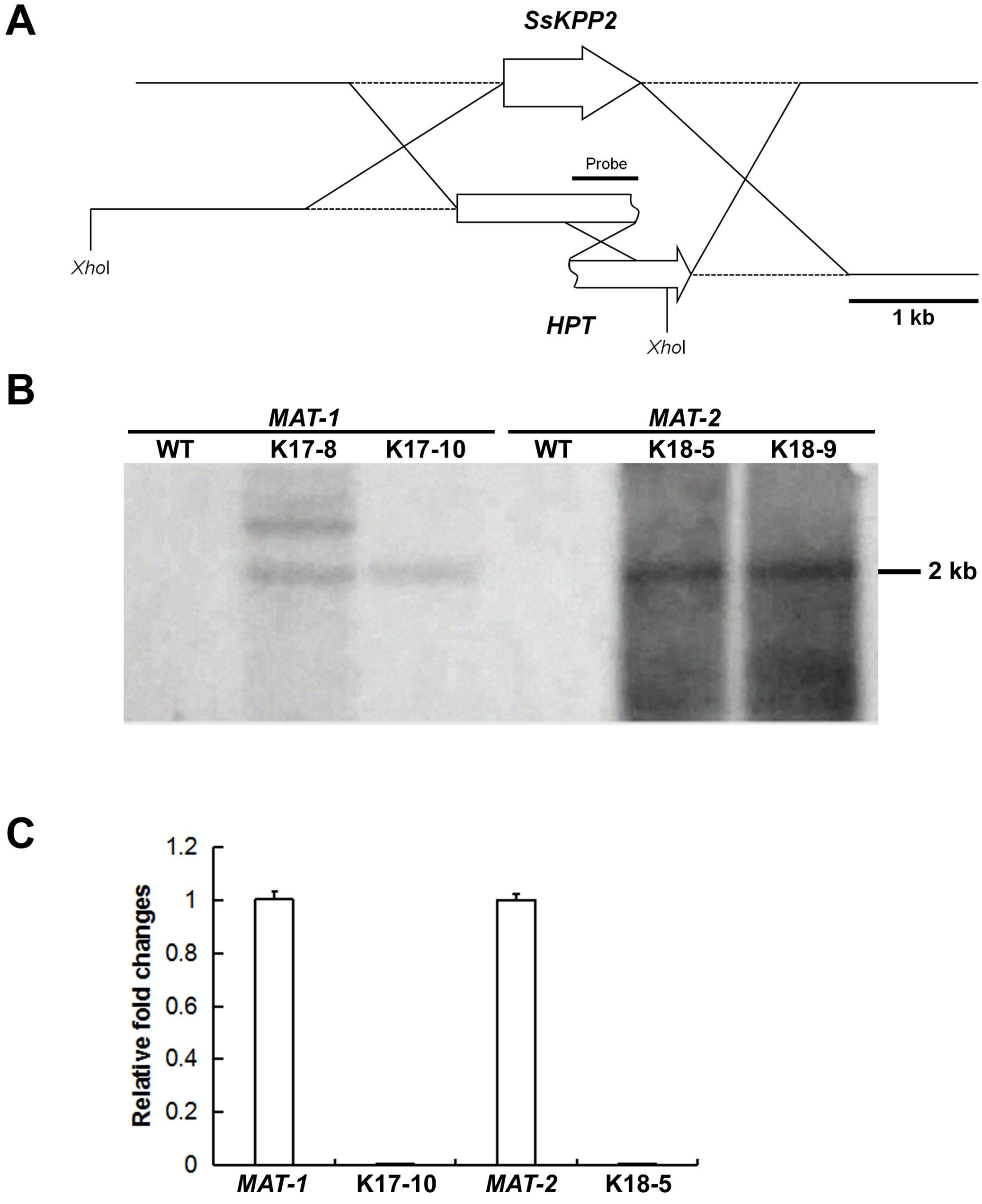
A
SsKPP2
Probe
XhoI
HPT
XhoI
1 kb
B
MAT-1
MAT-2
WT
K17-8
K17-10
WT
K18-5
K18-9
2 kb
C
Relative fold changes
1.2
1
0.8
0.6
0.4
0.2
0
MAT-1
K17-10
MAT-2
K18-5

Figure S2 Generation and verification of *tyna-1*Δ and *tyna-2*Δ mutants. (A) Schematic illustration of *S. scitamineum TYNA1 or TYNA2* locus (labelled as *TYNA1/2*), not draw to scale. Open bars represent coding region of targeted gene and dashed lines denote the homologous region constructed flanking the hygromycin-resistance cassette (*HPT*). The genomic fragment outside the homologous region was represented as solid line. The arrows denote to the position of the primers used to verify the transformants as shown in (B) and their DNA sequences were listed in Table S1. (B) Verification of *tyna-1*Δ or *tyna-2*Δ mutant by PCR amplification. Genomic DNA from the wild-type *MAT-1* or *MAT-2*, and the transformants in these two mating-type background (+ represents in *MAT-1* background, and - in *MAT-2* background) was PCR amplified using the primers as denoted in (A) to verify the deletion of *TYNA-1* or *TYNA-2* coding region. Amplification with the wild-type specific primers TYNA1/2-F+TYNA1/2-R, reflected the presence of intact gene *TYNA-1* or *TYNA-2* in *MAT-1* or *MAT-2* strains; while amplification with the wild-type locus primer TYNA1/2-F combined with *HPT* specific primer HPT-R, was dignostic as successful deletion of target gene. (C) The *tyna-1*Δ *or tyna-2Δ* mutants in the indicative mating-type background were confirmed by Semiquantitative RT-PCR using gene-specific primers as listed in Table S2. Relative gene expression level was calculated with -ΔΔCt method (Livak & Schmittgen, 2001) with *ACTIN* as internal control.

Figure S2

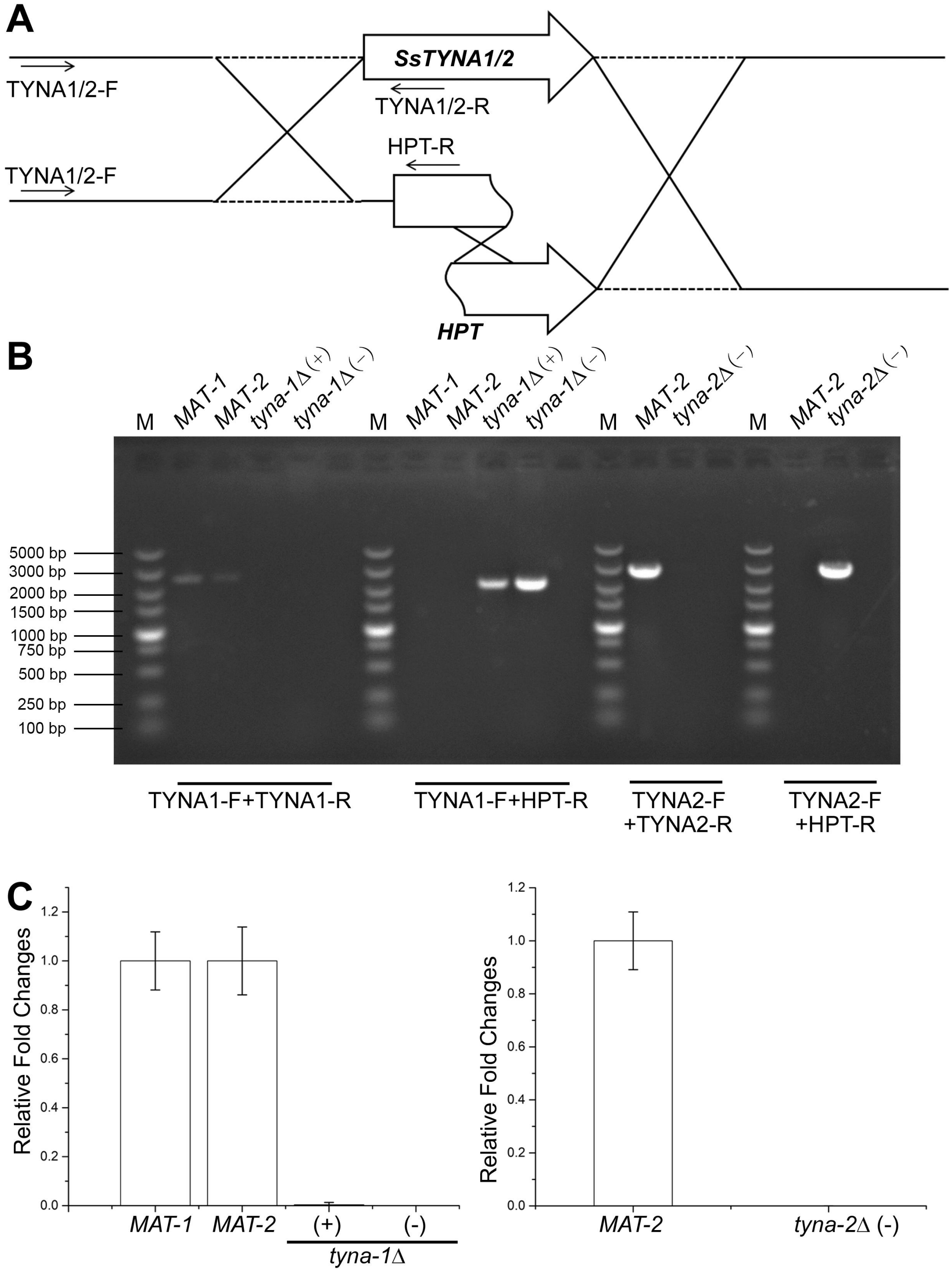
A
TYNA1/2-F
SsTYNA1/2
TYNA1/2-R
HPT-R
TYNA1/2-F
HPT
B
M
MAT-1
MAT-2
tyna-1Δ(+)
tyna-1Δ(−)
MAT-2
tyna-2Δ(−)
5000 bp
3000 bp
2000 bp
1500 bp
1000 bp
750 bp
500 bp
250 bp
100 bp
TYNA1-F+TYNA1-R
TYNA1-F+HPT-R
TYNA2-F +TYNA2-R
TYNA2-F +HPT-R
C
Relative Fold Changes
0.0
0.2
0.4
0.6
0.8
1.0
1.2
MAT-1
MAT-2
(+)
(-)
tyna-1Δ
tyna-2Δ (-)

Table S1. Selected genes for transcriptional profiling between WT and *sskpp2*Δ.

Gene number	Gene name	Sequences of qPCR primers
GenBank: CP010914.1 857085-857204	*MFA1*	MFA1-For: 5’-ATGCTTTCCATCTTTACCCAGA-3’
		MFA1-Rev: 5’-GTGCAGCTAGAGTAGCCAAG-3’
GenBank: LK056662.1 794925-795044	*MFA2*	MFA2-For: 5’-CGTCCAGGCCATTGTTTCT-3’
		MFA2-Rev: 5’-TAGGCCACGGTGCAGTA-3’
GenBank: CP010914.1 859744-860179	*PRA1*	PRA1-For: 5’-GGACGCTATCACCCAATCTTAC-3’
		PRA1-Rev: 5’-TCTCCAACATGGCAACACTC-3’
SPSC_01805	*PRA2*	PRA2-For: 5’-GAAGAGCCTCAGCCGTTATAC-3’
		PRA2-Rev: 5’-GGGTTCCCTTACTGAACCTTAG-3’
GenBank: CP010914.1 794193-795534	*bE1*	bE1-For: 5’-CCAACGACGAAAGCGCGACG -3’
		bE1-Rev: 5’-GACTCTCTGCGAGCGGGCAT-3’
SPSC_01827	*bE2*	bE2-For: 5’-CCCGACACTCAGCGCCAAGA-3’
		bE2-Rev: 5’-TCGACCTGCGTGCGTGAACA-3’
GenBank: CP010914.1 796524-797778	*bW1*	bW1-For: 5’-CGAGAAAGGCACACAACGTC-3’
		bW1-Rev: 5’-CACCTTTTGGGGAGTTCCGA-3’
SPSC_01826	*bW2*	bW2-For: 5’-TGTTGATGAGCCAGTGCCTT-3’
		bW2-Rev: 5’-AGTTCCGACTGGCTGAAGTG-3’
SSCI14340.1	*PRF1*	PRF1-For: 5’-GCCACCTCAGCCGTCTATCG-3’
		PRF1-Rev: 5’-ACTCGCAGTAGCCTTGCTCG-3’
SPSC_03059	*ARO8*	ARO8-For: 5’-CCTGGTGTTGCGTTCATTCC-3’
		ARO8-Rev: 5’-CAAGCTCGGGCATCGTCTTA-3’
SPSC_00463	*ARO9*	ARO9-For: 5’-TCCGCACGAACCATCCTAAC-3’
		ARO9-Rev: 5’-AGGTCGAATGAGTCGCCTTG-3’
SPSC_03401	*DC*	DC-For: 5’-TGGAGGACCATTGTTGACCG-3’
		DC-Rev: 5’-CATCGAAGGCTTCCGCAAAG-3’
SPSC_00335	*TYNA-1*	TYNA-1 For: 5’-ACCAGTCCAACGGTAAGCAG-3’
		TYNA-1 Rev: 5’-TCTCCCAGAAATCGTTGGGC-3’

SPSC_00449	*TYNA-2*	TYNA-2 For: 5’-AAGGTTAACTCTGCCGCTCC-3’
		TYNA-2 Rev: 5’-GCTAATACCATGCCTCGGCT-3’
SSCI56010.1	*BNA1*	BNA1-For: 5’-GTGTTTGCGAATCGAACGGG-3’
		BNA1-Rev: 5’-GGTGATTGACGAGGATGGCA-3’
SSCI63670.1	*BNA4-1*	BNA4-1 For: 5’-CCAAGCGGCTCCATGTTGCG-3’
		BNA4-1 Rev: 5’-TCTCATGCTCGCCTCGTCGC-3’
SPSC_06049	*BNA4-2*	BNA4-2 For: 5’-TGTGCATGTCTTGACGGTGA-3’
		BNA4-2 Rev: 5’-AATGGCTTCACGACTGGGAG-3’
SPSC_00684	*BNA5-2*	BNA5-2 For: 5’-CAGGAGGAAGCTAAAGAGGC −3’
		BNA5-2 Rev: 5’-CAACCCCCTCTGCTTTTCGG-3’
SSCI48940.1	*BNA7-1*	BNA7-1 For: 5’-ATTCGGTCTCAAGCCTGCTC-3’
		BNA7-1 Rev: 5’-AGAGCAGTTCATCGTCTCGC-3’
SSCI27490.1	*BNA7-2*	BNA7-2 For: 5’-CGAGGGTACGTACTTTGCCA-3’
		BNA7-2 Rev: 5’-GCGTCGGGAAAAATGTGGTG-3’
SPSC_04512	*ACTIN* *	Actin-For: 5’-CAGCTCGATGAAGGTCAAGAT-3’
		Actin-Rev: 5’-CACATCTGCTGGAAGGTAGAG-3’

* *ACTIN* gene is used as internal control.

Table S2 List of primers used for targeted gene deletion and verificatoin.

Name	Primer sequences	Description
pEX2-*HPT-For*	5’-GCAAGACCTGCCTGAAACCG-3’	Deletion construction
pEX2-*HPT-Rev*	5’-GGTCAAGACCAATGCGGAGC-3’	
Kpp2-1For	5’-GAGATTCCGATAGGCGAGAG-3’	
Kpp2-1Rev	5’-GCCTATCAGCAAGATCTCGATGCTGTCTTGTGAGATG-3’	
Kpp2-2For	5’-CATCTCACAAGACAGCATCGAGATCTTGCTGATAGGC-3’	
Kpp2-2Rev	5’-ACAATCATAAGGAAGCATCCTAATTCGGGGGATCTGGAT-3’	
Kpp2-3For	5’-ATCCAGATCCCCCGAATTAGGATGCTTCCTTATGATTGT-3’	
Kpp2-3Rev	5’-ATCCAGGGCTTCTTTGATAT-3’	

TYNA1-1For	5’-AGAAGCGTGCTACTCTATCAA-3’	
TYNA1-1Rev	5’-TTGCAAACTTTGGCTCTGTTCATCAGATCTTG CTGA-3’	
TYNA1-2For	5’-CTCTGTTCATCAGATCTTGCTGATAGGCAGG-3’	
TYNA1-2Rev	5’-TTTGCATTCCGATAATTCGGGGGATCTGGAT-3’	
TYNA1-3For	5’-TCCCCCGAATTATCGGAATGCAAACAAGCGT CATG-3’	
TYNA1-3Rev	5’-AGACGTCGCTTCTGCGGAAACGCT-3’	
TYNA2-1For	5’-TATCGCTCGTCCGCTTGATGAGTCG-3’	
TYNA2-1Rev	5’-TCAGCAAGATCTCATGGCGAACTTGTGGGTA ATGA-3’	
TYNA2-2For	5’-AAGTTCGCCATGAGATCTTGCTGATAGGCAG G-3’	
TYNA2-2Rev	5’-CATGACGATGCATAATTCGGGGGATCTGGAT-3’	
TYNA2-3For	5’-TCCCCCGAATTATGCATCGTCATGACTACATT CTC-3’	
TYNA2-3Rev	5’-TGATGGTGGCGAAGCTACTGGCG-3’	
Kpp2-For	5’-ACAGCAGTCCAACCAGTC-3’	Verification
Kpp2-Rev	5’-TTCTCAACCTTCTTCCTCTG-3’	
TYNA1-For	5’-GGTGTAGTTGTGGTGGATT-3’	
TYNA1-Rev	5’-CTTGACGGTGAACGAAGA-3’	
TYNA2-For	5’-GCTCTCCAACAGTCTCAAG-3’	
TYNA2-Rev	5’-ACCTCACAGTCTCGCATA-3’	
HPT-For	5’-CGCCATCGTCTTCTTCTT-3’	
HPT-Rev	5’-CGTCAGGACATTGTTGGA-3’	
Kpp2-Probe-For	5’-ATCAGTCGATGGGAGGGAGGTAG-3’	Southern blot probe
Kpp2-Probe-Rev	5’-TGAGGCTTGTGCGGCAGCGGGCC-3’	

TableS3 List of primers used for qRT-PCR

Gene number	Gene name	Sequences of qPCR primers
GenBank: CP010914.1 857085-857204	*MFA1*	MFA1-For: 5’-ATGCTTTCCATCTTTACCCAGA-3’
		MFA1-Rev: 5’-GTGCAGCTAGAGTAGCCAAG-3’
GenBank:		MFA2-For: 5’-CGTCCAGGCCATTGTTTCT-3’

LK056662.1 794925-795044	*MFA2*	MFA2-Rev: 5’-TAGGCCACGGTGCAGTA-3’
GenBank: CP010914.1 859744-860179	*PRA1*	PRA1-For: 5’-GGACGCTATCACCCAATCTTAC-3’
		PRA1-Rev: 5’-TCTCCAACATGGCAACACTC-3’
SPSC_01805	*PRA2*	PRA2-For: 5’-GAAGAGCCTCAGCCGTTATAC-3’
		PRA2-Rev: 5’-GGGTTCCCTTACTGAACCTTAG-3’
GenBank: CP010914.1 794193-795534	*bE1*	bE1-For: 5’-CCAACGACGAAAGCGCGACG -3’
		bE1-Rev: 5’-GACTCTCTGCGAGCGGGCAT-3’
SPSC_01827	*bE2*	bE2-For: 5’-CCCGACACTCAGCGCCAAGA-3’
		bE2-Rev: 5’-TCGACCTGCGTGCGTGAACA-3’
GenBank: CP010914.1 796524-797778	*bW1*	bW1-For: 5’-CGAGAAAGGCACACAACGTC-3’
		bW1-Rev: 5’-CACCTTTTGGGGAGTTCCGA-3’
SPSC_01826	*bW2*	bW2-For: 5’-TGTTGATGAGCCAGTGCCTT-3’
		bW2-Rev: 5’-AGTTCCGACTGGCTGAAGTG-3’
SPSC_03059	*ARO8*	ARO8-For: 5’-CCTGGTGTTGCGTTCATTCC-3’
		ARO8-Rev: 5’-CAAGCTCGGGCATCGTCTTA-3’
SPSC_00463	*ARO9*	ARO9-For: 5’-TCCGCACGAACCATCCTAAC-3’
		ARO9-Rev: 5’-AGGTCGAATGAGTCGCCTTG-3’
SPSC_03401	*DC*	DC-For: 5’-TGGAGGACCATTGTTGACCG-3’
		DC-Rev: 5’-CATCGAAGGCTTCCGCAAAG-3’
SPSC_00335	*TYNA-1*	TYNA-1 For: 5’-ACCAGTCCAACGGTAAGCAG-3’
		TYNA-1 Rev: 5’-TCTCCCAGAAATCGTTGGGC-3’
SPSC_00449	*TYNA-2*	TYNA-2 For: 5’-AAGGTTAACTCTGCCGCTCC-3’
		TYNA-2 Rev: 5’-GCTAATACCATGCCTCGGCT-3’
SSCI56010.1	*BNA1*	BNA1-For: 5’-GTGTTTGCGAATCGAACGGG-3’
		BNA1-Rev: 5’-GGTGATTGACGAGGATGGCA-3’
SSCI63670.1	*BNA4-1*	BNA4-1 For: 5’-CCAAGCGGCTCCATGTTGCG-3’
		BNA4-1 Rev: 5’-TCTCATGCTCGCCTCGTCGC-3’
	BNA4-2	BNA4-2 For: 5’-TGTGCATGTCTTGACGGTGA-3’

SPSC_06049		BNA4-2 Rev: 5’-AATGGCTTCACGACTGGGAG-3’
SPSC_00684	*BNA5-2*	BNA5-2 For: 5’-CAGGAGGAAGCTAAAGAGGC -3’
		BNA5-2 Rev: 5’-CAACCCCCTCTGCTTTTCGG-3’
SSCI48940.1	*BNA7-1*	BNA7-1 For: 5’-ATTCGGTCTCAAGCCTGCTC-3’
		BNA7-1 Rev: 5’-AGAGCAGTTCATCGTCTCGC-3’
SSCI27490.1	*BNA7-2*	BNA7-2 For: 5’-CGAGGGTACGTACTTTGCCA-3’
		BNA7-2 Rev: 5’-GCGTCGGGAAAAATGTGGTG-3’
SPSC_04512	*ACTIN*	Actin-For: 5’-CAGCTCGATGAAGGTCAAGAT-3’
		Actin-Rev: 5’-CACATCTGCTGGAAGGTAGAG-3’
